# Linking dissemination and implementation science to Learning Health Systems: Opportunities for Clinical and Translational Science Award institutions

**DOI:** 10.1017/cts.2020.15

**Published:** 2020-02-26

**Authors:** Nancy M. Bennett, Elissa Orlando, Paul Meissner

**Affiliations:** 1 Clinical and Translational Science Institute, University of Rochester School of Medicine and Dentistry, Rochester, NY, USA; 2 Montefiore Medical Center, Albert Einstein College of Medicine, Bronx, NY 10467, USA

**Keywords:** Clinical Translation Science Awards, Learning Health Systems, institutional website profile, key informant interviews, recommendations

## Abstract

Learning Health Systems (LHS) iteratively implement and evaluate health improvement projects. Dissemination and implementation (D&I) science is the study of evidence-based practices in real-world settings, a critical tool for LHS. This paper explores intersections between LHS and D&I science in Clinical and Translational Science Awards (CTSAs) institutions and identifies critical components of collaboration. We conducted website scans of 34 CTSAs and their home institutions that had Dissemination, Implementation, and Knowledge Translation (DIKT) Workgroup members. We identified linkages between CTSAs and their institutions’ LHS. We interviewed six CTSA leaders experienced in LHS and D&I sciences. Nearly half of CTSAs identified an LHS structure on their websites, but only one-third indicates CTSA involvement in these efforts. Interviewees identified key components for successful integration of LHS and D&I sciences: leadership, infrastructure, balance between rigor and efficiency, and aligned incentives. The need for research integration in LHS, to improve evaluation and increase knowledge, is an emerging opportunity for D&I scientists and CTSAs. CTSAs that are engaged in D&I science can introduce and/or expand the role of D&I science in LHS. Collaboration between CTSAs and clinical leaders could result in strengthened relationships between clinical and research enterprises, effective and efficient health care delivery, and improved health.

## Introduction

**Fig. 1.**
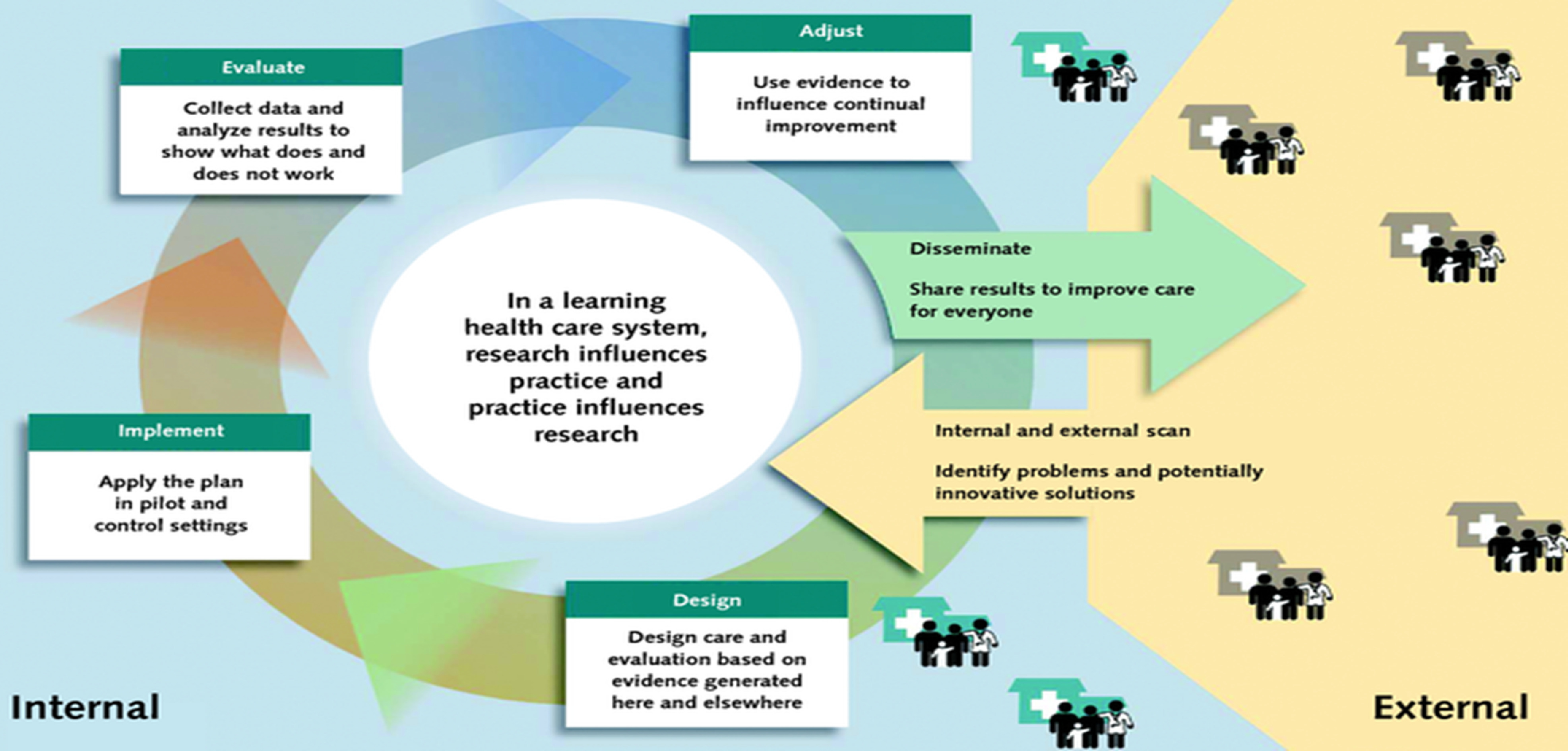
Learning Health Systems (LHS) figure from Sarah Greene, Robert Reid, and Eric Larson, implementing the LHS: from concept to action. https://annals.org/aim/fullarticle/1305510/implementing-learning-health-system-from-concept-action.

An approach to improving population health is the Learning Health System (LHS), defined and described in an IOM report in 2013 [1]. An LHS is an approach that iteratively implements and evaluates projects that result in measurable improvements in population health and efficient use of health care and community resources. The goal is a cycle in which scalable and replicable interventions are piloted, studied, and implemented (Fig. 1). Clinical and Translational Science Awards (CTSAs), developed by the NIH to enhance the role of research in improving health, can be helpful in providing research support to an LHS [2].

“Dissemination and implementation research seeks to understand how to best apply scientific advances in the real world, by focusing on pushing the evidence-based knowledge base out into routine use” [3]. This is essentially the last step in translational science and is a critical tool in an LHS. Leppin et al., in a companion article in this issue, define and discuss the distinctions between the sciences of dissemination, implementation, and translation and point out that dissemination science and implementation science are each consistent with the ideals and function of an LHS [4].

This *Special Communication* explores the relationship between LHS and dissemination and implementation (D&I) sciences in institutions with CTSAs. It is not a comprehensive review or a scientific study, but rather is designed to generate discussion among CTSAs and their parent institutions, by identifying critical components of each discipline and their integration. Recognizing that CTSA engagement with D&I science is still in the early adoption period and that CTSA sites encounter difficulty in distinguishing their activities and scientific inquiry, we considered D&I sciences together in relationship to both CTSAs and LHS structures and functions.

## Methods

To better understand the intersections among LHS, D&I science, and CTSAs, we searched the institutional and CTSA websites of the 34 CTSAs represented on the Dissemination, Implementation, and Knowledge Translation (DIKT) Workgroup of the CTSA Consortium. We chose this sample because we felt the findings would be reflective of D&I-engaged CTSAs. The terms “Learning Health System” and “Learning Healthcare System” were searched in the search boxes of the CTSA hub websites and of the corresponding institutions’ home pages. We explored whether there were references to an LHS on the institution’s website and if the reference existed, whether there were website linkages between the reference to an LHS and the CTSA hub. There were challenges related to this method. In many cases, the linkages were not readily apparent and required extensive web searching.

To further characterize the nature of intersections between CTSAs and LHS, we conducted key informant interviews of selected leaders at six CTSAs. Interviewees were chosen to represent a diversity of perspective and experience with both LHS and D&I science. Interviewees were provided with a series of questions prior to the interview. At the conclusion of each interview, subjects were asked to provide one or more recommendations for CTSA hubs exploring connections among LHS, D&I science, and CTSAs.

## Results

Table 1 shows the distribution of sample institutions (*n* = 34) as to how their websites referred to LHS and their CTSA: 16 (47%) institutions referred to an LHS; 11 of those (32% of total, but 69% of LHS institutions) linked to their CTSA, while in 5 (15% total, 31% of LHS institutions), there was no link found between the LHS and the CTSA. In 18 (53%), no mention of an LHS was found on the institutional or CTSA websites.

**Table 1.** Institutions with Learning Health Systems (LHS) approaches and links to Clinical and Translational Science Awards (CTSAs).

CTSA Dissemination, Implementation, and Knowledge Translation (DIKT) Workgroup Institutions (*n* = 34)			
Referred to LHS	LHS/CTSA link	LHS/no link to CTSA	No mention LHS
16 (47%)	11 (32%)	5 (15%)	18 (53%)

Interviews were conducted with key informants. While all the institutions represented by the key informants were considering ways to enhance the integration of research and clinical priorities, only a few institutions have developed infrastructure to do so. Integration between LHS efforts and the CTSA was most highly developed at institutions where there was institutional leadership for an LHS with an understanding of the value of D&I science and of CTSA infrastructure. In some cases, leadership grew from CTSA efforts and intentionally developed integrated approaches. In other cases, integration between the CTSA hub and the LHS is still evolving with distributed models such as working groups and committees.

D&I science was rarely fully integrated with LHS efforts. Interviewees pointed out that the most successful approaches to LHS involved rapid evaluation of interventions addressing critical health care system issues [5] and that D&I science often was conducted in a more leisurely manner addressing issues of more importance to investigators than the health care system. Even in institutions with infrastructure to perform rapid evaluation of clinical interventions, D&I science was not fully integrated into the LHS.

Several CTSAs supported D&I education and training opportunities that are bringing LHS approaches and D&I science together, such as the Agency for Healthcare Research and Quality (AHRQ) and the Patient-Centered Outcomes Research Institute (PCORI) grants to support training in patient-centered outcomes research. These programs appear to have developed a strong model for joint clinical and research mentorship of trainees, which may result in enhanced clinical and research partnerships.

Only one interviewee described an integrated LHS program which evolved from a CTSA D&I program. All interviewees were enthusiastic about the potential synergy of CTSA D&I efforts and LHS, but most said their institutions were still defining and exploring ways to create this synergy.

Several essential components, often referenced in D&I models, were consistently identified through the interviews.

(1) Leadership: There must be at least one senior institutional leader who identifies the potential impact of using an LHS approach and who understands the value of research in the process. A data-driven quality improvement culture forms a foundation for an LHS approach.

Example: In several successful institutions, leadership came from either the Chief Executive Officer or Chief Medical Officer, who often had a research background and recognized the value of including rigorous evaluation in improving health-system interventions.

(2) Infrastructure: There must be informatics capacity and analytic resources guided by a shared clinical and research oversight group that can identify critical clinical initiatives and priorities. For an LHS to truly be a system where, “research influences practice and practice influences research,” both investigators and clinicians must be engaged leaders.

Example: In several institutions, there was a designated “data shop” to implement rapid analyses and studies to evaluate clinical interventions. Projects were generally chosen by clinical leaders with methodological input from investigators. Institutions had a variety of leadership structures to support the assessment of priorities.

(3) Balance: While the interviewees believed that D&I science can lend methodologic rigor to an LHS, they pointed out the need to balance rigor with efficiency. D&I science can provide evaluation and support for continuous learning, but interviewees who work in clinical enterprises call out the need for expeditious and efficient evaluation and rapid cycle studies. Training in D&I science, delivered through CTSAs, may advance the expertise needed to meet a variety of needs in research and operational settings, but must be balanced by deep commitment to efficiently address the most pressing clinical problems.

Example: All institutions developing successful programs pointed to the necessity of bringing clinicians and investigators together to better understand common values and goals. In addition, several institutions have developed innovative training programs which explicitly link clinical leaders to researchers as co-mentors for trainees. This co-mentorship can lead to greater collaboration, understanding, and balance in approaches to health improvement.

(4) Alignment of incentives: Several interviewees mentioned a misalignment of incentives between researchers and clinical leaders. These tensions between academic advancement and health care system needs were described as hindrances to development of collaborative models. Leadership must recognize this and find ways to bridge and align incentives that is acceptable to both perspectives.

Example: Several institutions have developed departments addressing population health, which include expertise in D&I science, and units dedicated to doing rapid clinical studies to support the LHS. Investigators are identifying rapid evaluation procedures that may be linked to more fully developed D&I studies. As promotion and tenure guidelines are expanded to value team science and population health improvement, it is likely that this discrepancy in incentives will be lessened.

## Conclusions

Over the last 13 years, the enhanced and centralized access to training and tools for clinical and translational research, supported by the CTSA program, has increased the efficiency of research, but delays in implementation and dissemination persist, stimulating awareness and heightened interest in D&I sciences and the development, within CTSAs, of expertise in this growing field.

Since the description of an LHS by the IOM, many institutions have adopted this approach to improving population health. It is a valuable approach to quality and population health improvement employed by almost 50% of the institutions involved in the CTSA Consortium DIKT Workgroup. And yet, only two-thirds of these institutions note, on their websites, involvement of their CTSAs in furthering these efforts, despite D&I sciences capacity in their CTSA.

Our initial explorations of intersections between LHS and CTSA D&I activities suggest a variety of lessons for both clinical leaders and D&I scientists. First and foremost is the requirement for clinical leadership to identify priorities for LHS approaches and D&I sciences. Second is the need for infrastructure, especially data analytic capacity, that includes D&I scientists in the development of LHS. Third is the requirement that institutions balance rigor and efficiency in developing LHS approaches to population health improvement. Fourth is the necessity to recognize the conflicting incentives of research and clinical enterprises in academic medical centers and to form bridges to develop common goals and incentives.

This exploration is limited to CTSA hubs that participate in the DIKT Workgroup of the CTSA Consortium. It only describes a little more than half of the CTSA hub institutions and does not include the many other medical centers in the country addressing population health. However, these are the institutions that seemed most likely to have linkages between their population health efforts and their CTSAs, due to their interest and expertise in D&I sciences.

Our commentary only skims the surface of the innovations occurring in institutions and CTSAs across the country and should be expanded by a more complete and comprehensive study. Sharing the variety of approaches to integrating research, in particular D&I sciences, into LHS is imperative to move the field forward and to ensure the best institutional structures to support efforts to improve health and health care. CTSAs can play a critical role in developing expertise and infrastructure for D&I sciences that will advance LHS.
